# Supplementary material for: Magnetic Bifunctional Ru-Enzyme Catalyst Allows for Sustainable Conversion of Cellulose Derivative to D-Sorbitol
Source: Nanomaterials (Basel). 2025 May 15;15(10):740. doi: 10.3390/nano15100740 (PMC12114484; doi:10.3390/nano15100740)
Supplement: Supplementary file 1 [file nanomaterials-15-00740-s001.zip › nanomaterials-3596374-supplementary.pdf]

Supplementary materials

Materials

Iron (III) chloride hexahydrate (99%) ($FeCl_3 \times 6\ H_2O$), iron (II) chloride tetrahydrate (99%) ($FeCl_2 \times 4H_2O$), ammonium hydroxide solution (25%), citrate buffer solution (CBS buffer), phosphate buffered saline (PBS buffer), and sodium tripolyphosphate (TPP) were purchased from Nevareaktiv (Russia) and used without purification. Ruthenium (IV) hydroxychloride ($Ru(OH)Cl_3$) was purchased from Sigma-Aldrich and used without purification. Cellulase (Cel) lyophilized powder (EC 3.2.1.8, 50 U/mg) was purchased from Biopreparat (Russia) and used as received. Chitosan middle-viscous, insoluble matter <1%, 400KDa and N-(3-dimethylamiopropyl)-N'-ethyl carbodiimide hydrochloride (EDC, 99%) were obtained from FLUKA, BioChemika (Japan). N-Hydroxysuccinimide (NHS, 98%) was purchased from Acros Organics (China). 3,5-Dinitrosalicylic acid (DNS) was purchased from Pallav (India). Carboxymethylcellulose (CMC) sodium salt was purchased from Juning Fortune Biotech (China).

Synthesis of MNA

In a typical experiment, 15 mL of the $FeCl_3 \times 6H_2O$ solution (2.162 g in 15 mL of distilled water) and 15 mL of the $FeCl_2 \times 4H_2O$ solution (0.792 g in 15 mL of distilled water) were mixed in a jacketed glass reactor. The mixture was stirred for 15 min at a rate of 400 min^{-1} at room temperature, after which the temperature in the reactor was increased to 65 °C. The mixture was stirred for another 15 min. After that, 10 mL of the aqueous ammonia solution (25%) was added dropwise at a rate of 2 mL /min followed by stirring for another 15 min. The MNA yield is 95% of the calculated amount.

Modification of MNA with chitosan and tripolyphosphate

To cover the MNA with chitosan, in a typical experiment, 10 mL of chitosan solution (0.1 g in 10 mL of 2M acetic acid) was added to the MNA reaction mixture (without separation of MNA) and stirred for 15 minutes. After that, MNA-CS was separated from the reaction mixture using a rare earth magnet. For CS cross-linking on the MNA surface, the MNA were stirred for 1 h in a solution of TPP (0.05 g in 50 mL of distilled water), after which the MNA were washed five times, separated with a rare earth magnet, and dried in air at 20 °C for 24 h. The resulting sample was designated as MNA-CSP where CS stands for chitosan and P stands for TPP. The MNA-CSP yield is 93% of the calculated amount.

Synthesis of MNA-CSP-Ru

In a typical experiment, ruthenium hydroxochloride solution was prepared by stirring 0.077 g $Ru(OH)Cl_3$ for 15 minutes in 15 mL of tetrahydrofuran, 2.5 mL of methanol, and 2.5 mL of distilled water. Then the mixture was heated to 60 °C and MNA-CSP particles (1 g) were added. The mixture was left to stir for 24 hours at 60 °C to ensure coordination of Ru ions with chitosan amino groups. After that, the particles were washed several times with distilled water and extracted using a rare earth magnet. Next, a $NaBH_4$ solution was prepared (0.06 g in 50 mL of distilled water) in the ice bath. The $NaBH_4$ solution (25 mL) was mixed with the particles in the ice bath, stirred for 10 min, after which the remaining 25 mL of the $NaBH_4$ solution was added dropwise and stirred for another 10 min. After that, the particles were washed several times with distilled water, extracted using a rare earth magnet, and dried in air. According to the X-ray fluorescent spectroscopy, the Ru content is 3.02 wt.%. The MNA-CSP-Ru yield is 94% of the calculated amount.

Synthesis of MNA-CSP-Cel and MNA-CSP-Ru-Cel by immobilization of Cel

For the covalent attachment of Cel on the support, we used EDC and NHS. When these reagents are added and kept in the reaction mixture for 12 h, a stable NHS ester is formed on the surface of Cel. This resulted in the formation of a durable amide bond with the NH_2 groups on the support surface [1,2].

In a typical experiment, the dry MNA-CSP/MNA-CSP-Ru sample (1.0 g) was added to 0.1 g of EDC, 0.04 g of NHS, and 50 mg of Cel dissolved in 25 mL of citrate buffer (pH 5.0) and stirred for 12 h. Then the biocatalyst (MNA-CSP-Cel/MNA-CSP-Ru-Cel) was magnetically separated, washed five times with 50 mL of water each, and dried at 20 °C for 24 h.

The enzyme content varied from 1.25 to 10 wt.%, i.e., in terms of Cel concentration in the reaction from 0.05 to 0.4 mg/mL. The activity of native and immobilized Cel was assessed by the amount of D-glucose produced in 30 min at pH 5 and 50 °C.

The effectiveness of immobilization was determined by the concentration of Cel in the fractions of the supernatant (as well as before immobilization) using the method proposed by Bradford [3]. For the MNA-CSP-Cel/MNA-CSP-Ru-Cel samples, immobilization was complete, since no Cel was detected in the supernatant.

Catalytic efficiency of native and immobilized Cel

Cellulase activity was determined according to the original reducing sugar analysis method (DNS assay) [4,5]. In a typical experiment, 5 mg of native or immobilized cellulase (0.1 g of MNA-CSP-Cel) was incubated with 0.25% (w/v) of CMC in 25 mL of the 0.1 M sodium citrate buffer with pH 5.0. The reaction mixture was kept under constant stirring for 30 min at 50 ° C. The D-glucose released was measured using the UV-5 spectrophotometer (UV/VIS Mettler Toledo) at 540 nm by the DNS method and expressed as glucose equivalent using a standard calibration curve.

Relative activity of Cel was calculated according to the following equation:

Relative activity (%) = (Activity of immobilized Cel/Activity of native Cel)×100%

Upong changes of temperature or pH, the other conditions were kept the same with the 2 mg/mL CMC concentration.

To determine kinetics, the initial reaction rates for both native and immobilized Cel, were obtained at varied concentrations of CMC (1.91-11.45 mM) in the sodium citrate buffer at pH 5.0 and 50 °C. Based on these data, the maximum reaction rate (V_{max}) and the Michaelis-Menten constant (K_m) were computed using the Lineweaver-Burk analysis [6].

The storage stability analysis has been performed. Free and immobilized Cel was mixed in 0.1M sodium citrate buffer, pH 5.0 at 4 °C for a period of 49 days and the assay was performed to calculate the relative activity at an interval of 7 days. The control (100%) was observed as activity of cellulase after first day and residual activity was evaluated through it.

Catalytic properties of MNA-CSP-Ru

Hydrogenation of D-glucose was performed in a 100 mL stainless autoclave (Parr, Series 5000 Multiple Reactor) under vigorous stirring (1000 rpm). In a typical experiment, MNA-CSP-Ru (0.1 g) was mixed with 50 mL of 0.1M D-glucose solution in water (pH 7.0) and the reactor was purged with hydrogen four times to remove air. The reactor was heated to 100 °C and then kept at the hydrogen pressure of 4 MPa. The total reaction time is 120 min. Samples of the reaction mixture were taken for analysis regularly and analyzed by the high-performance liquid-phase chromatography (HPLC, Liquid Chromatograph Chromatek-Kristall 2014, equipped with a RI detector and a Maisch GmbH column ReproGelCa (8×300 mm). The eluent was water with a flow rate of 1 cm^3 min^{-1}. The column was kept at 80 °C by a column heater.

The D-glucose conversion and D-sorbitol selectivity equations are shown below:

D-glucose conversion, % = (mols of D-glucose $_{reacted}$/mols D-glucose $_{initial}$)×100%

Sorbitol selectivity, % = (mols of D-sorbitol/mols of D-glucose $_{reacted}$)×100%

MNA-CSP-Ru-Cel is a multicomponent catalytic system. To exclude the influence of individual components of the catalyst, we conducted hydrogenation of D-glucose to D-sorbitol with MNA-CSP and MNA-CSP-Cel. The results showed that these samples do not exhibit catalytic activity in this process.

Catalytic properties MNA-CSP-Ru-Cel

All one-pot cascade processes were performed in a 100 mL stainless steel autoclave (Parr reactor (Series 5000 Multiple Reactor) under vigorous stirring (1000 rpm). In a typical experiment, MNA-CSP-Ru-Cel (0.1 г) was incubated with 0.25% (w/v) CMC in 25 mL of a sodium citrate buffer with pH 7.0 and the reactor was purged with hydrogen four times to remove air. The reactor was heated to 70 °C and kept at the hydrogen pressure of 4 MPa for 5 h, 7.5 h, or 10 h. Samples of the reaction mixture were taken for analysis regularly and analyzed by HPLC.

The CMC conversion, and the D-sorbitol yield were calculated by the following equations [7]:

CMC conversion (%) = [(initial CMC weight - CMC weight after the reaction)/initial CMC weight]×100%

D-sorbitol yield (%) = (moles of D-sorbitol/moles of D-glucose units in CMC)×100%

For reusability experiments, MNA-CSP-Ru-Cel was separated with a rare earth magnet after each catalytic reaction and triple washed using a sodium citrate buffer at pH 7 in order to remove the remaining substrate that could be attached to the support surface. Finally, the nanobiocatalyst was added to the next reaction mixture.

The hot filtration test has been carried out in optimized conditions to confirm heterogeneity of the catalyst in hydrogenation of D-glucose to D-sorbitol. MNA-CSP-Ru-Cel was removed at the reaction temperature after 1 h (at half the product yield) using a magnet. The filtrate was then allowed to continue the reaction under the same conditions in the absence of the catalyst. The filtrate was analyzed at certain times intervals. It was found that after the catalyst removal, no product was formed, indicating the heterogeneous character of this process.

Characterization

A Spectroscan Max spectrometer was used to determine the Ru content in MNA-CSP-Ru by X-ray fluorescence analysis (XFA).

Atomic absorption spectroscopy (AAS) to study Ru leaching in the reaction solution was carried out with Atomic absorption spectrometer "MGA-915" equipped with electrothermal atomization and correction of non-selective spectral noise based on the use of the High Frequency Zeeman background effect (Lumex, Russia). It is also supplied with a lamp with a hollow cathode per elemental Ru.

Nitrogen adsorption measurements were carried out at liquid nitrogen temperature on an Beckman Coulter TM SA 3100TM (Coulter Corporation, USA) using a sample preparation device Beckman Coulter TM SA-PREPTM (Coulter Corporation, USA). Samples were degassed at 100 °C in vacuum. The total surface area was estimated by the Brunauer–Emmett–Teller (BET) method, while the pore size distribution was determined by the Barrett–Joyner–Halenda (BJH) method using desorption.

Transmission electron microscopy (TEM) and high resolution (HRTEM) images as well as energy scanning TEM dispersive spectroscopy (EDS) were acquired on Osiris at 200 kV. The samples were prepared by placing a drop of the material suspension on the carbon coated TEM grid.

Magnetization measurements of the samples were carried out in a home-built vibrating sample magnetometer (VSM) with accuracy better than ±0.01 emu/g, which allows measuring bulk and powder samples of 0.01-150 mg in the temperature range of 80-1000 K and magnetic field of 0-2.5 T.

X-ray photoelectron spectroscopy (XPS) data were obtained using MgKα (hυ = 1253.6 eV) radiation with the ES-2403 spectrometer (Institute for Analytical Instrumentation of the RAS, St. Petersburg, Russia) equipped with an energy analyzer PHOIBOS 100-MCD5 (SPECS, Berlin, Germany) and a X-ray source XR-50 (SPECS, Berlin, Germany). All the data were acquired at the X-ray power of 250W. Survey spectra were recorded at an energy step of 0.5 eV with an analyzer pass energy of 40 eV. Samples were allowed to outgas for 180 min before analysis and were stable during the examination. The data analysis was performed with CasaXPS.

Thermal gravimetric analysis (TGA) was performed on TG IRIS209 F1 NETZSCH placing ~5-10 mg of the powder in aluminum pans. The experiments were carried out upon heating to 600 °C at a rate of 10.0 °C/min.

X-ray powder diffraction (XRD) was carryout out on Rigaku MiniFlex600 (Rigaku Corporation, Japan) using CuKα-radiation (40kB, 15mA, and Ni-K_β filter) in the angle range 2θ = 10 – 90 ° with the step of 0.02° and the rate of 0.5°/min.

Results

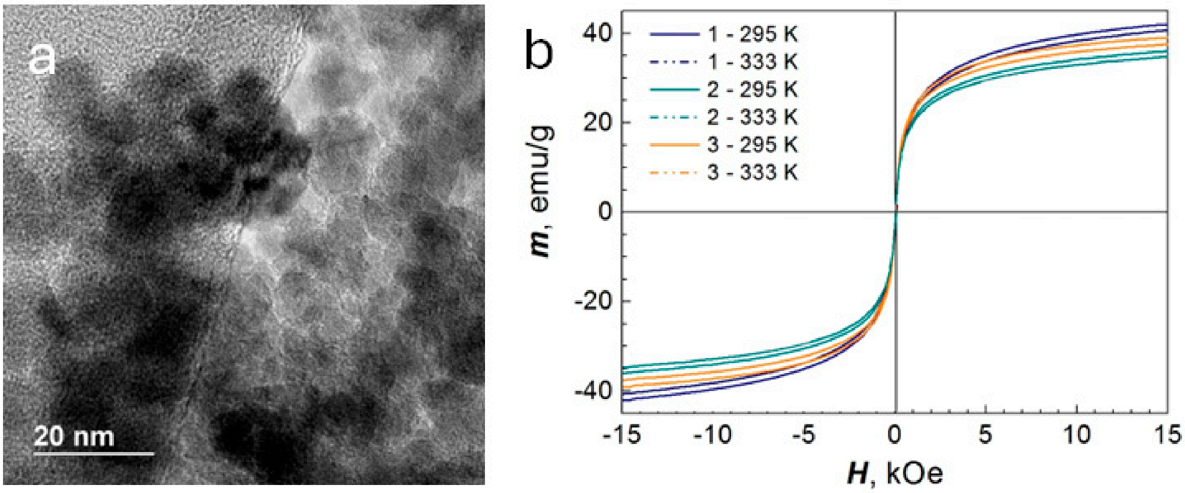

Figure S1. HRTEM image of MNA (a) and hysteresis loops (b) of MNA (1), MNA-CSP (2), and MNA-CSP-Ru (3).

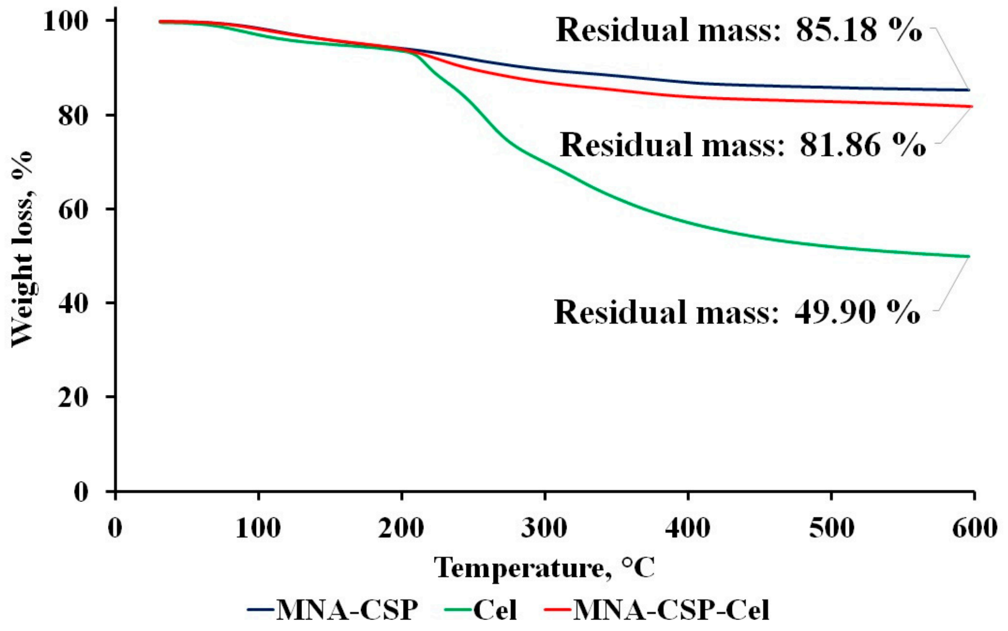

Figure S2. TGA curves for Cel, MNA-CSP, and MNA-CSP-Cel.

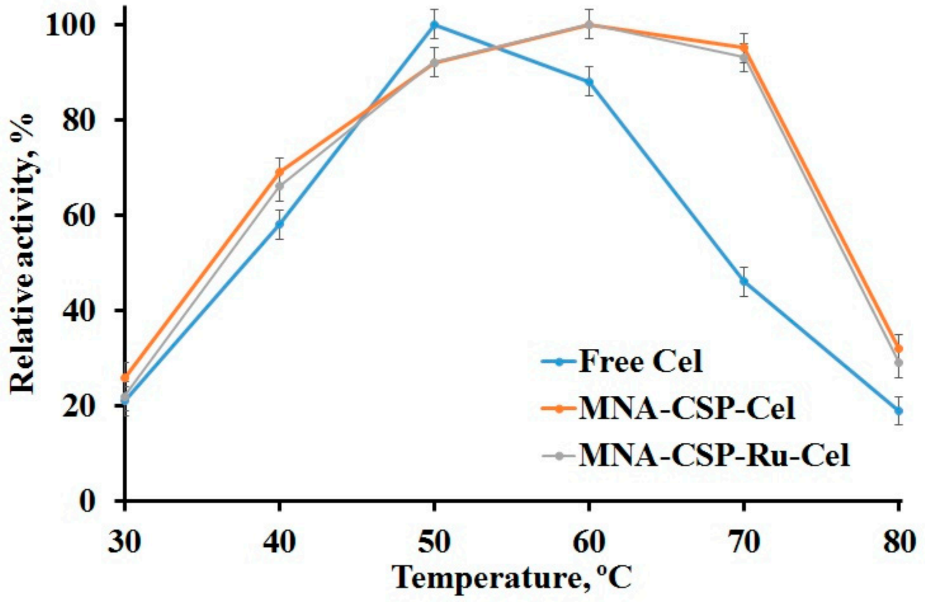

Figure S3. Dependence of the enzyme activity on temperature in hydrolysis of CMC to D-glucose for native Cel, MNA-CSP-Cel and MNA-CSP-Ru-Cel. Reaction conditions: 5 mg of free or immobilized Cel (0.1 g of MNA-CSP-Cel/MNA-CS-Ru-Cel), 25 mL of 0.25% (w/v) of CMC with pH 5.0. The reaction mixture was kept under constant stirring for 30 min.

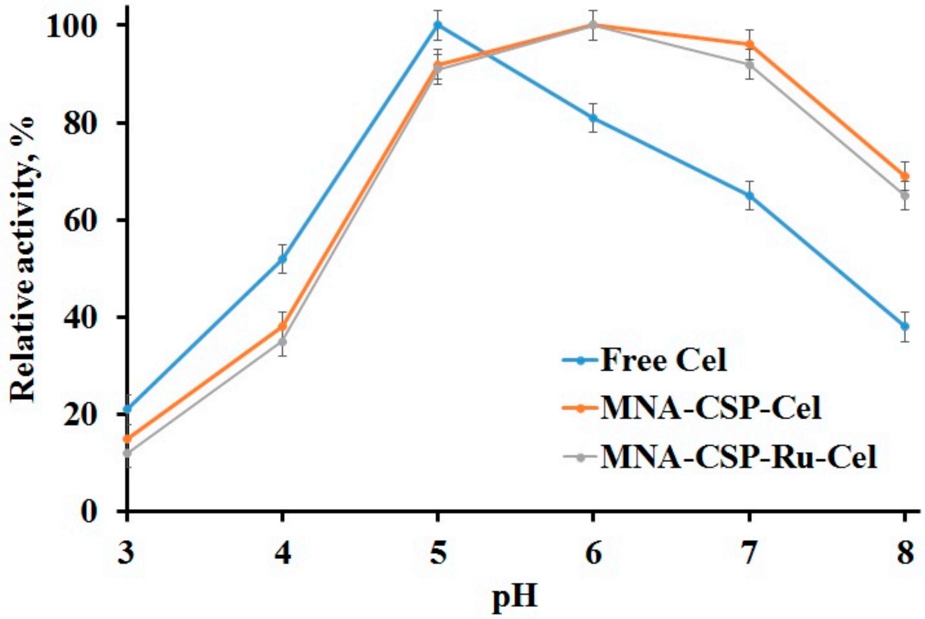

Figure S4. Dependence of the enzyme activity on the pH value in hydrolysis of CMC to D-glucose for native Cel, MNA-CSP-Cel and MNA-CSP-Ru-Cel. Reaction conditions: 5 mg of free or immobilized Cel (0.1 g of MNA-CSP-Cel/MNA-CS-Ru-Cel), 25 mL of 0.25% (w/v) of CMC. The reaction mixture was kept under constant stirring for 30 min at 50 ° C.

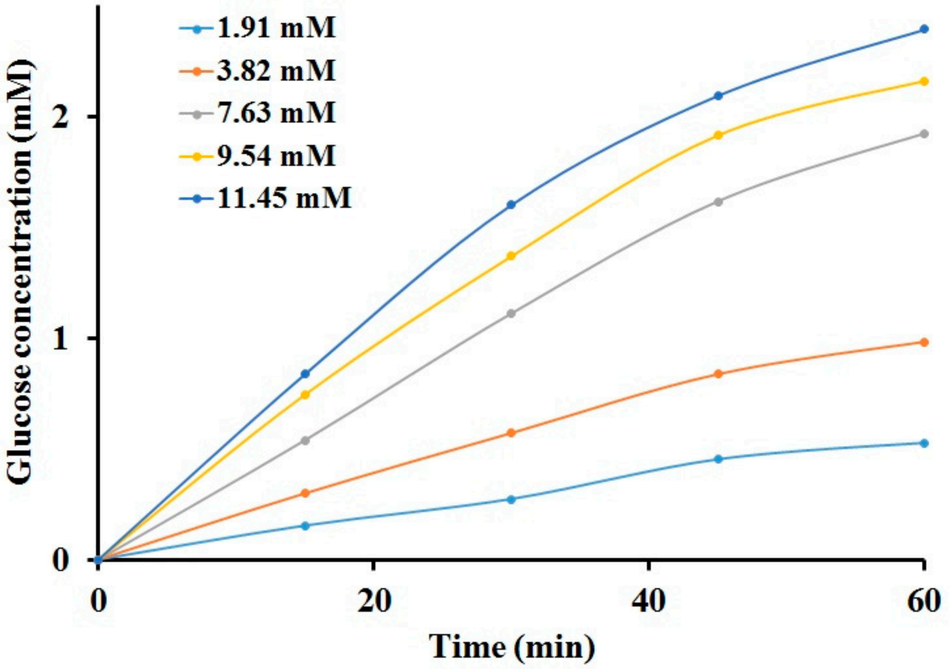

Figure S5. Kinetic curves for native Cel. Reaction conditions: 5 mg of free Cel, 25 mL of the CMC solution with pH 5.0. The reaction mixture was kept under constant stirring at 50 ° C.

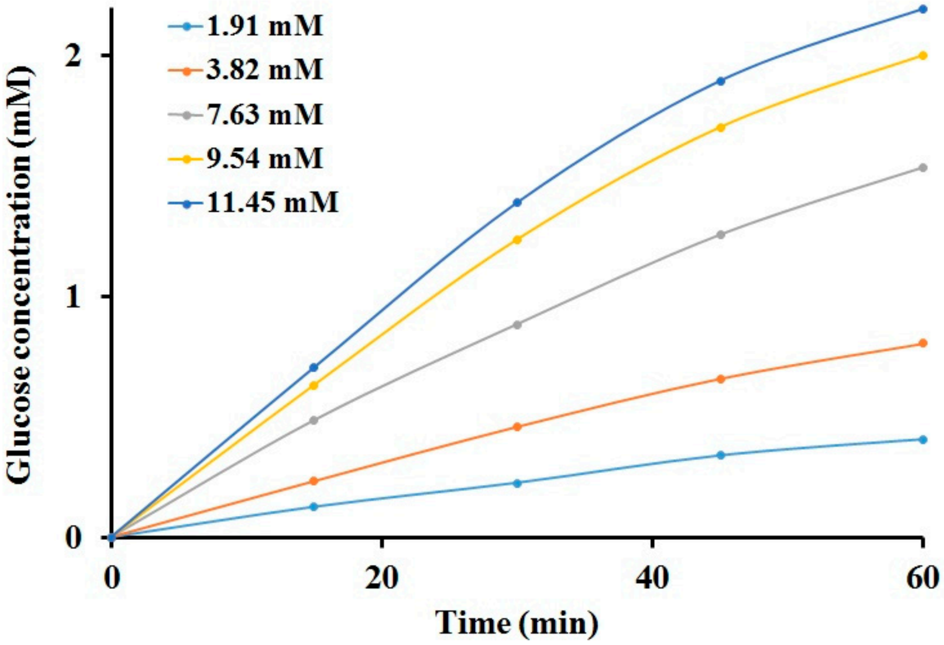

Figure S6. Kinetic curves for MNA-CSP-Cel. Reaction conditions: 0.1 g of MNA-CSP-Cel, 25 mL of the CMC solution with pH 5.0. The reaction mixture was kept under constant stirring at 50 ° C.

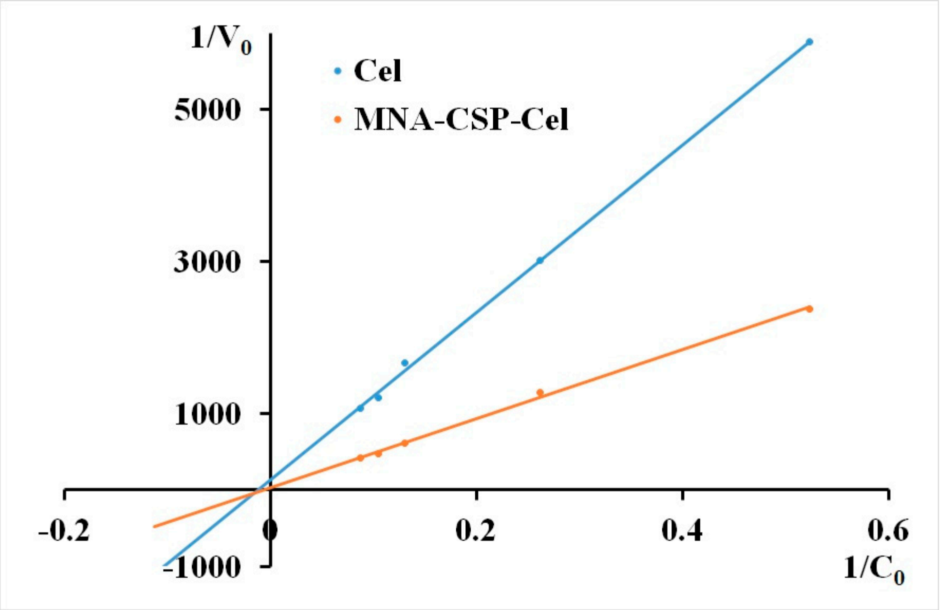

Figure S7. Lineweaver-Burk plot for native Cel and MNA-CSP-Cel.

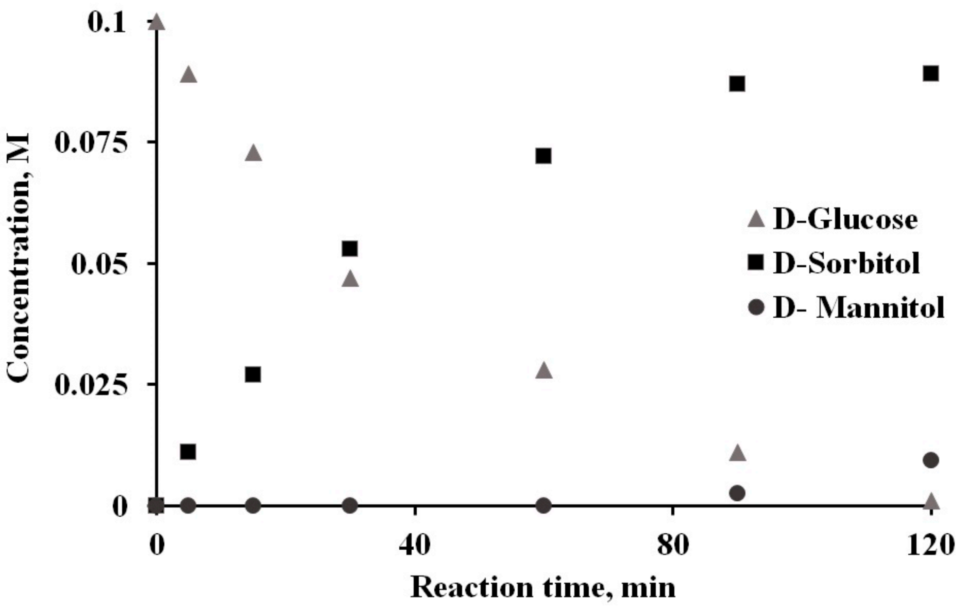

Figure S8. Kinetic profile of D-glucose conversion on MNA-CSP-Ru (5% Ru).
Reaction conditions: temperature of 100°C, H_2 pressure of 4 MPa, reaction volume of 50 mL, pH 7.0, D-glucose concentration of 0.1 M, catalyst amount of 0.1 g, reaction time of 120 min. Stirring rate 1000 rpm.

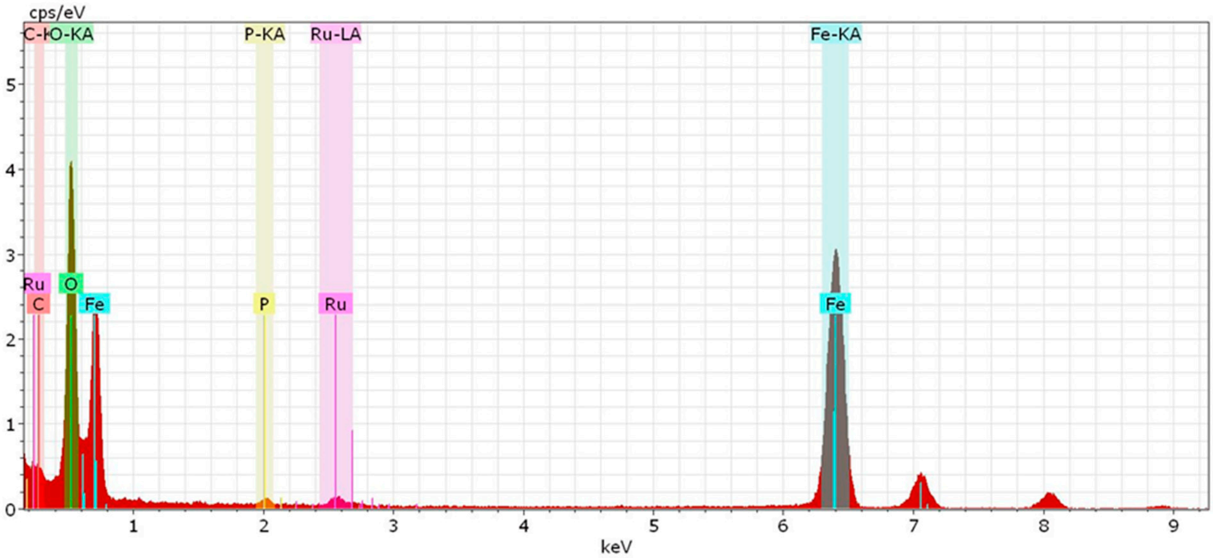

Figure S9. EDS spectrum of MNA-CS-Ru-Cel.

Table S1. Composition of MNA-CSP-Ru-Cel obtained from the survey XPS spectrum.

Element content, wt.%	O	C	N	Fe	P	Ru	Na	Cl
Before reaction	41.46	13.32	1.26	32.01	4.17	5.49	0.60	1.69
After the first catalytic cycle	41.75	14.44	1.33	32.88	2.12	6.07	0.43	0.98

Table S2. Deconvolution of the high-resolution XPS spectrum of MNA-CSP-Ru-Cel (see Figure 5b).

Name	Pos., eV	FWHM, eV	% Area
Ru $3d_{5/2}$	281.70	2.27	23.97
Ru $3d_{3/2}$	285.87	2.27	15.95
C 1s	284.70	2.55	44.35
C1s C=0	287.67	2.99	15.73

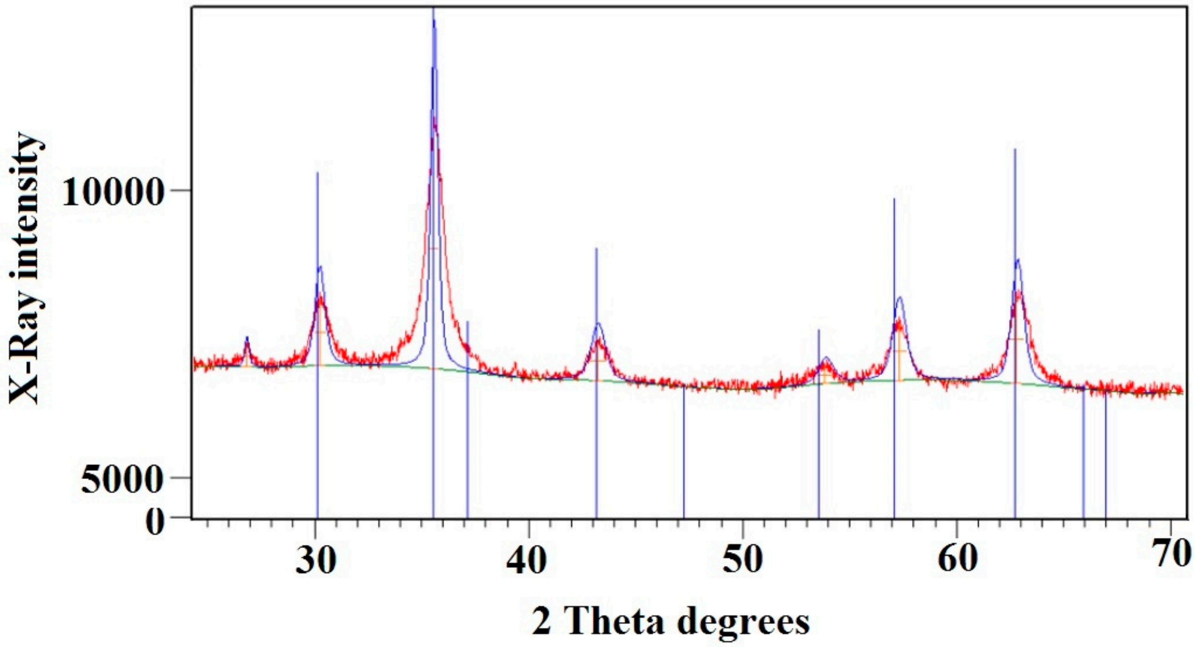

Figure S10 XRD pattern of MNA-CSP-Ru-Cel. All reflections in the pattern match spinel [8].

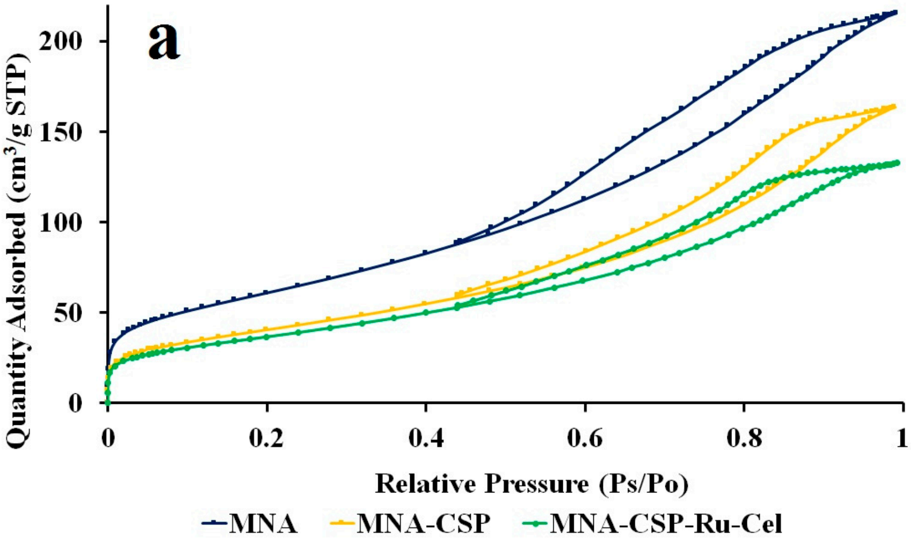

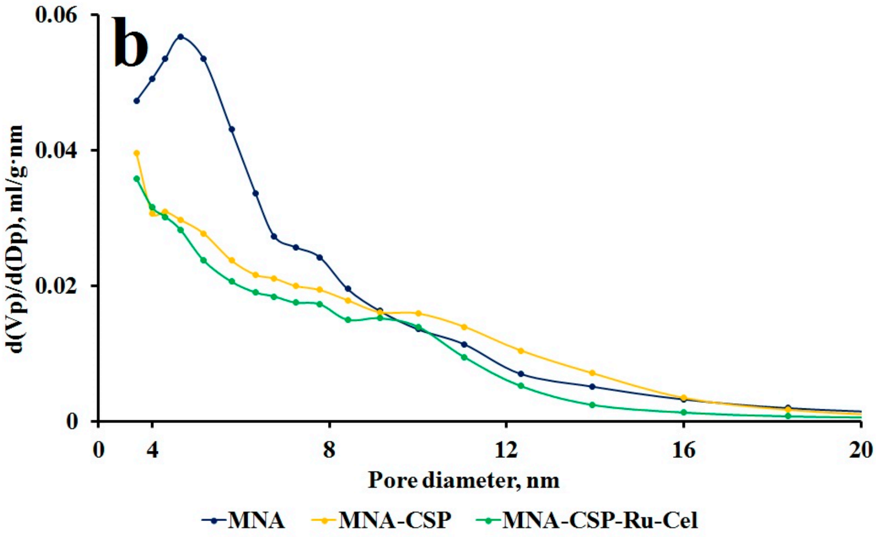

Figure S11. Liquid nitrogen adsorption-desorption isotherms (a) and pore size distributions (b) for MNA, MNA-CSP, and MNA-CSP-Ru-Cel.

Table S3. BET surface areas, pore volumes, and pores sizes for MNA, MNA-CSP, and MNA-CSP-Ru-Cel.

Sample notation	BET surface area, m^2/g	BET pore volume, cm^3/g
MNA	220	0.3335
MNA-CSP	150	0.3091
MNA-CSP-Ru-Cel	132	0.2050

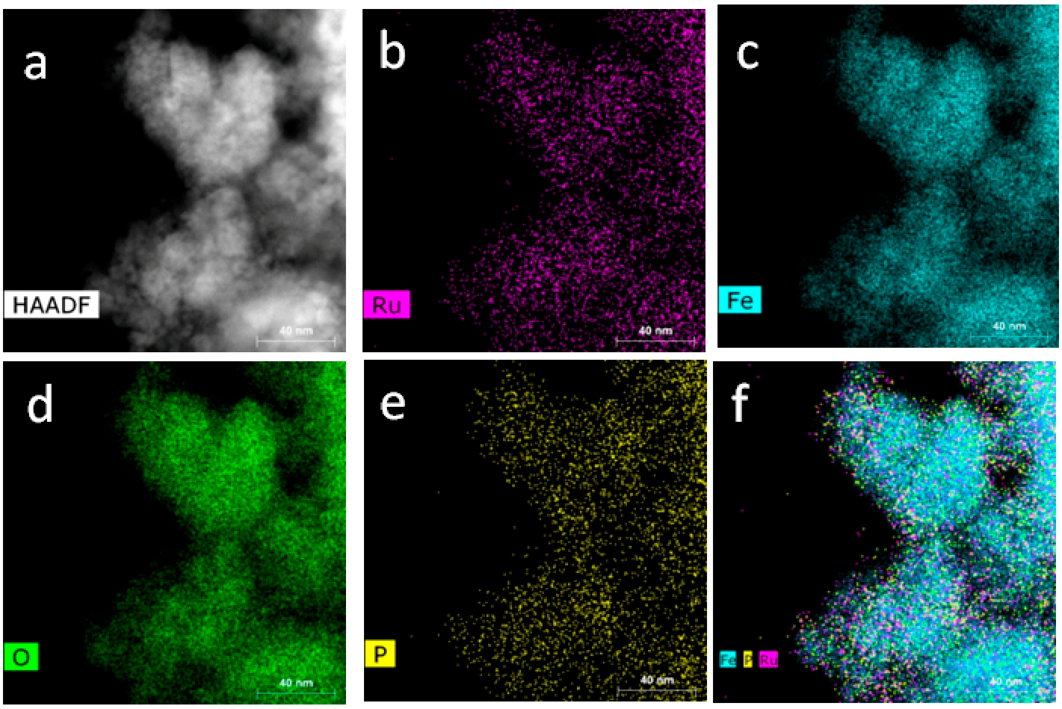

Figure S12. HAADF image (a), and STEM EDS maps of Ru (b), Fe (c), O (d), and P (e) in MNA-CSP-Ru. Superposition of the Fe, P, and Ru maps is shown in (f).

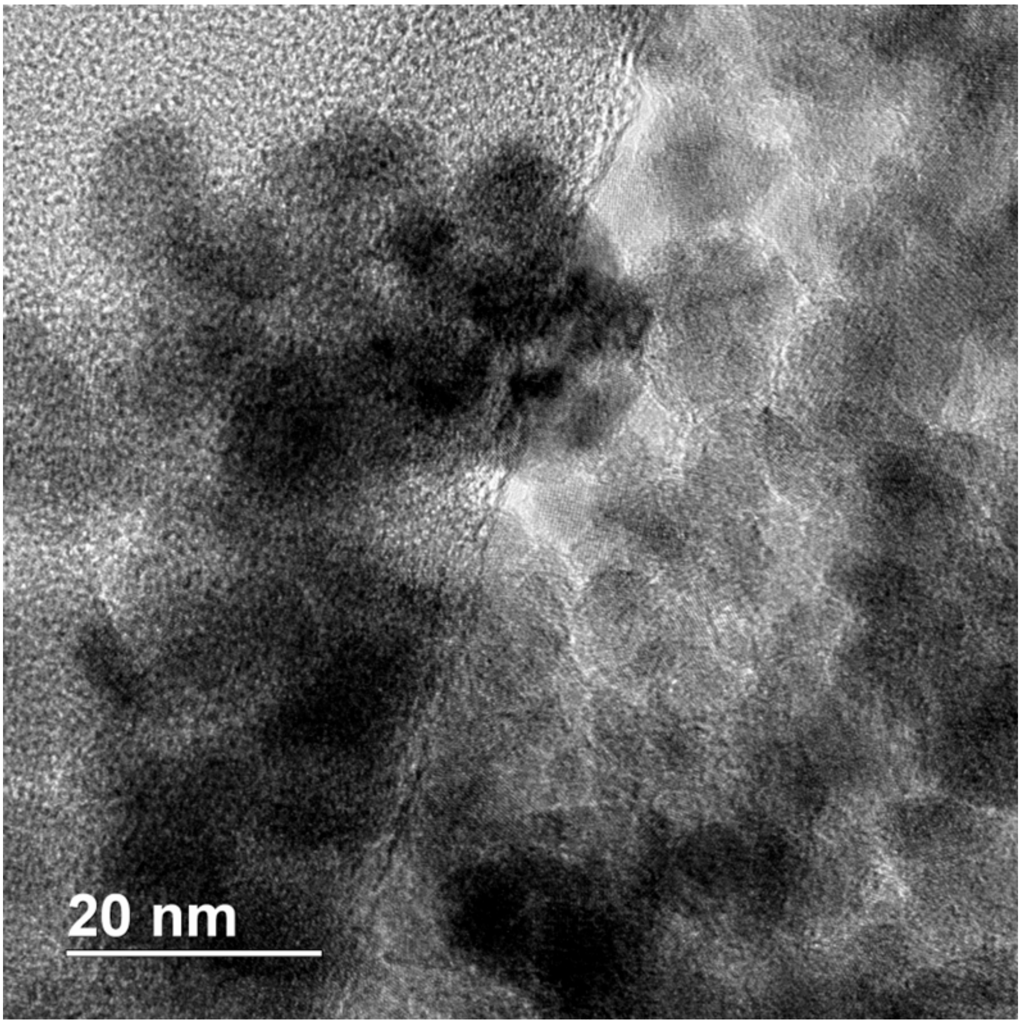

Figure S13. HRTEM image of MNA-CSP-Ru.

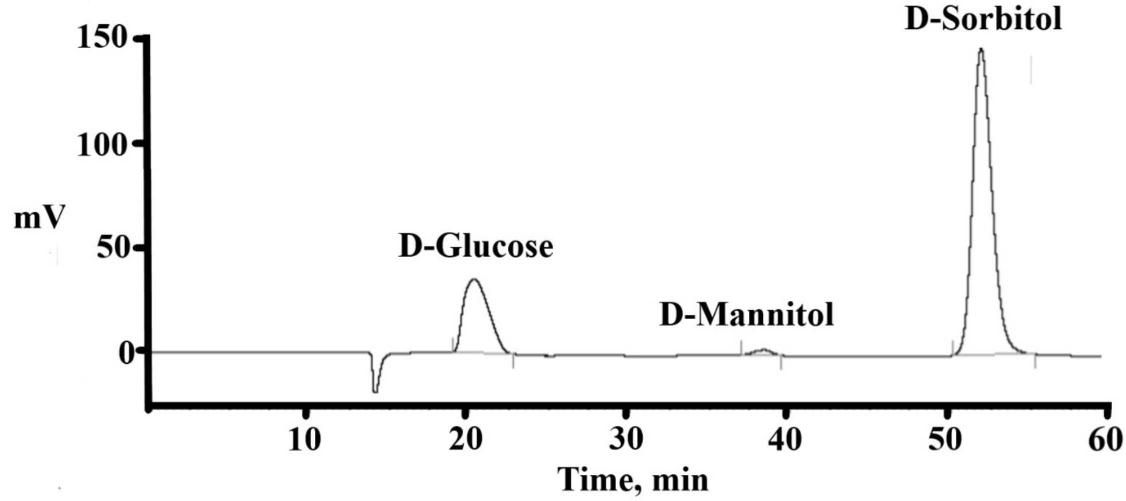

Figure S14. Chromatogram of the reaction mixture obtained with MNA-CSP-Ru-Cel after 7.5 h (the D-glucose conversion of 70.9% with the selectivity to D-sorbitol of 98.8%.)

The carbon mass balance (typical for biomass related processes) was calculated for the CMC conversion for the 7.5 h reaction time. The CMC sodium salt (0.0625 g) was loaded into the reactor and the mass of carbon there was 0.0258 g. The composition of the reaction mixture after 7.5 hours was 29.1% of D-glucose, 70% of D-sorbitol, and 0.09% of D-mannitol. The mass of

carbon in these compounds was 0.0066 g, 0.0162 g, and 0.0021 g, respectively (a total of 0.0249 g). This was 96.5% of the initial carbon amount.

References

1. Ahmad, R.; Khare, S.K. Immobilization of Aspergillus niger cellulase on multiwall carbon nanotubes for cellulose hydrolysis. *Bioresour. Technol.* **2018**, *252*, 72-75, doi:10.1016/j.biortech.2017.12.082.
2. Jiang, J.; Gong, X.; Li, T.; Huang, J.; Zhou, N.; Jia, X. Immobilized Cellulase on NH2-MIL-88(Fe) and Its Performance as a Biocatalyst. *Appl. Biochem. Biotechnol.* **2024**, *196*, 4745-4758, doi:10.1007/s12010-023-04759-5.
3. Bradford, M.M. A rapid and sensitive method for the quantitation of microgram quantities of protein utilizing the principle of protein-dye binding. *Anal. Biochem.* **1976**, *72*, 248, doi:10.1016/0003-2697(76)90527-3.
4. Miller, G.L. Dinitrosalicylic acid reagent for determination of reducing sugar. *Anal. Chem.* **1959**, *31*, 426, doi:10.1021/ac60147a030.
5. Vasilescu, C.; Marc, S.; Hulka, I.; Paul, C. Enhancement of the Catalytic Performance and Operational Stability of Sol-Gel-Entrapped Cellulase by Tailoring the Matrix Structure and Properties. *Gels* **2022**, *8*, 626, doi:10.3390/gels8100626.
6. Javid, A.; Amiri, H.; Kafrani, A.T.; Rismani-Yazdi, H. Post-hydrolysis of cellulose oligomers by cellulase immobilized on chitosan-grafted magnetic nanoparticles: A key stage of butanol production from waste textile. *Int. J. Biol. Macromol.* **2022**, *207*, 324-332, doi:10.1016/j.ijbiomac.2022.03.013.
7. Li, Z.; Liu, Y.; Liu, C.; Wu, S.; Wei, W. Direct conversion of cellulose into sorbitol catalyzed by a bifunctional catalyst. *Bioresour. Technol.* **2019**, *274*, 190-197, doi:10.1016/j.biortech.2018.11.089.
8. Manaenkov, O.V.; Mann, J.J.; Kislitza, O.V.; Losovyj, Y.; Stein, B.D.; Morgan, D.G.; Pink, M.; Lependina, O.L.; Shifrina, Z.B.; Matveeva, V.G.; et al. Ru-Containing Magnetically Recoverable Catalysts: A Sustainable Pathway from Cellulose to Ethylene and Propylene Glycols. *ACS Appl. Mater. Interfaces* **2016**, *8*, 21285-21293, doi:10.1021/acsami.6b05096.
